# VEGF-A and neuropilin 1 (NRP1) shape axon projections in the developing CNS via dual roles in neurons and blood vessels

**DOI:** 10.1242/dev.151621

**Published:** 2017-07-01

**Authors:** Lynda Erskine, Urielle François, Laura Denti, Andy Joyce, Miguel Tillo, Freyja Bruce, Neil Vargesson, Christiana Ruhrberg

**Affiliations:** 1 School of Medicine, Medical Sciences and Nutrition, Institute of Medical Sciences, University of Aberdeen, Aberdeen AB25 2ZD, UK; 2 UCL Institute of Ophthalmology, University College London, 11-43 Bath Street, London EC1V 9EL, UK

**Keywords:** Retinal ganglion cell, Optic chiasm, Blood vessel, Axon guidance, Neuropilin, VEGF-A

## Abstract

Visual information is relayed from the eye to the brain via retinal ganglion cell (RGC) axons. Mice lacking NRP1 or NRP1-binding VEGF-A isoforms have defective RGC axon organisation alongside brain vascular defects. It is not known whether axonal defects are caused exclusively by defective VEGF-A signalling in RGCs or are exacerbated by abnormal vascular morphology. Targeted NRP1 ablation in RGCs with a *Brn3b^Cre^* knock-in allele reduced axonal midline crossing at the optic chiasm and optic tract fasciculation. In contrast, *Tie2-Cre*-mediated endothelial NRP1 ablation induced axon exclusion zones in the optic tracts without impairing axon crossing. Similar defects were observed in *Vegfa^120/120^* and *Vegfa^188/188^* mice, which have vascular defects as a result of their expression of single VEGF-A isoforms. Ectopic midline vascularisation in endothelial *Nrp1* and *Vegfa^188/188^* mutants caused additional axonal exclusion zones within the chiasm. As *in vitro* and *in vivo* assays demonstrated that vessels do not repel axons, abnormally large or ectopically positioned vessels are likely to present physical obstacles to axon growth. We conclude that proper axonal wiring during brain development depends on the precise molecular control of neurovascular co-patterning.

## Abstract (precis)

**Summary:** NRP1 plays a dual role in retinal ganglion cells and in vascular endothelial cells to organise axons along the optic pathway between the mouse retina and diencephalon.

## INTRODUCTION

The coordinated development of neuronal and vascular networks is a prerequisite for normal nervous system architecture and function. Beginning around embryonic day (E) 10 in mice, vessels sprout from a perineural vascular plexus into the brain and spinal cord and extend towards the ventricular surface, where they branch and anastomose to form a subventricular vascular plexus (Tata et al., 2015; Wälchli et al., 2015). This vascular network then expands and remodels to meet the metabolic demand of the growing neural tissue. Accurate axon pathfinding therefore requires that avascular areas are established to provide spaces for axon growth, or that axons activate mechanisms that enable them to navigate through an environment populated by vessels.

The vascular endothelial growth factor VEGF-A has essential roles in vascular and neuronal patterning in the developing central nervous system (CNS) (Tata et al., 2015). VEGF-A is produced as a collection of alternatively spliced isoforms, with the major murine isoforms consisting of 120, 164 or 188 amino acids. Owing to the presence or absence of heparin-binding domains, these isoforms differ in their affinity for extracellular matrix components and thereby form chemoattractant gradients that regulate vascular morphogenesis (Ruhrberg et al., 2002). The isoforms additionally differ in their receptor-binding properties (Tata et al., 2015). All VEGF-A isoforms bind with high affinity to KDR (FLK1, VEGFR2) and FLT1 (VEGFR1). VEGF_164_ and VEGF_188_, but not VEGF_120_, also bind with high affinity to neuropilin 1 (NRP1) (Tillo et al., 2015), which is expressed by both neurons and blood vessels (Fantin et al., 2013).

In vascular endothelial cells, NRP1 acts as a VEGF-A receptor to promote arteriogenesis and postnatal angiogenesis (Fantin et al., 2014; Gelfand et al., 2014; Lanahan et al., 2013). Additionally, NRP1 has VEGF-A-independent roles in extracellular matrix and TGFβ signalling during postnatal angiogenesis (Aspalter et al., 2015; Fantin et al., 2015; Raimondi et al., 2014). Although the specific pathway regulated by NRP1 for brain angiogenesis in the embryo has not yet been identified, it is mediated neither by VEGF-A nor by SEMA3A signalling (Fantin et al., 2014; Gelfand et al., 2014; Vieira et al., 2007), but is likely to involve TGFβ signalling (Hirota et al., 2015).

In neurons, NRP1 acts either as a receptor for VEGF_164_ and VEGF_188_ or as a receptor for class 3 semaphorins (Tata et al., 2015). As a VEGF-A receptor, NRP1 regulates migration, axon guidance and survival in specific subpopulations of neurons (Cariboni et al., 2011; Erskine et al., 2011; Schwarz et al., 2004; Tillo et al., 2015). For example, we previously demonstrated that retinal ganglion cells (RGCs) express NRP1 to sense chemoattractive VEGF-A signals that are essential for contralateral axon growth across the optic chiasm (Erskine et al., 2011).

In mice, the majority of RGC axons project contralaterally at the optic chiasm (Erskine and Herrera, 2014). Loss of NRP1 or NRP1-binding VEGF-A isoforms increases the proportion of RGC axons projecting ipsilaterally at the expense of contralateral projections, and axon organisation in the optic tracts is perturbed (Erskine et al., 2011; Tillo et al., 2015). In contrast, the optic pathway develops normally in mice lacking semaphorin signalling through NRPs (Erskine et al., 2011). Although expression studies and *in vitro* experiments have shown that contralateral growth at the optic chiasm can be explained by VEGF-A acting directly on NRP1-expressing contralateral RGC axons (Erskine et al., 2011), it is not known whether defective vascular patterning compounds the optic pathway defects.

Here, we have generated complementary mouse mutants lacking NRP1 in vascular endothelial cells versus RGCs and examined these as well as VEGF-A isoform mutants to determine the relative contribution of neural and vascular guidance pathways to RGC axon patterning. Our results demonstrate that NRP1 is required autonomously in RGCs for midline guidance at the optic chiasm, but that endothelial NRP1 as well as VEGF-A isoforms contribute to RGC axon organisation through roles in blood vessel morphogenesis.

## RESULTS

### NRP1 is essential for both optic pathway and vascular development

**Fig. 1.**
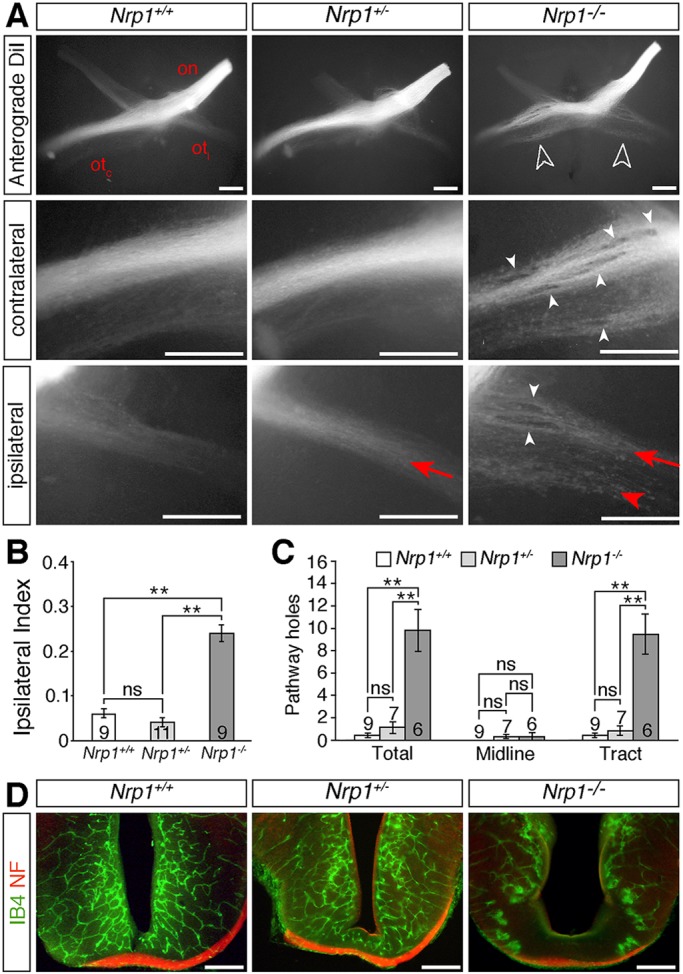
**NRP1 is essential for normal development of the optic pathway.** (A) Whole-mount view of RGC axons at the optic chiasm and contralateral and ipsilateral optic tracts in E14.5 *Nrp1* wild-type (*Nrp1^+/+^*), heterozygous (*Nrp1^+/−^*) and null (*Nrp1^−/−^*) littermates, labelled anterogradely from one eye with DiI. Ventral view (top panels) or lateral view (middle and bottom panels), anterior up; on, optic nerve; ot_c_, contralateral optic tract; ot_i_, ipsilateral optic tract. White unfilled arrowheads indicate the increased proportion of ipsilaterally projecting RGC axons in mutants, white arrowheads indicate axonal exclusion zones, red arrows the normal position and organisation of the ipsilateral projection, red arrowhead ipsilateral optic tract defasciculation in mutants. (B,C) Ipsilateral index (B) and total number of exclusion zones (holes) at the optic chiasm (midline) and in the contralateral optic tract (C) in E14.5 *Nrp1* wild-type, heterozygous and homozygous mutant littermates (mean±s.e.m.). **P<0.01; ns, not significant (one-way ANOVA with post-hoc Tukey). Numbers on bars indicate the number of embryos analysed for each genotype. (D) Coronal sections through the optic chiasm of E14.5 *Nrp1* wild-type, heterozygous and homozygous mutant littermates labelled with isolectin B4 (IB4) to visualise blood vessels (green) and antibodies against neurofilaments (NF) to visualise nerves (red). Scale bars: 250 µm.

NRP1 is expressed by developing RGC axons (Erskine et al., 2011) and nascent vessels as they extend into and within the developing brain (Fantin et al., 2013). We therefore investigated whether NRP1 regulates neurovascular co-patterning in the optic pathway. Anterograde DiI labelling confirmed our previous finding (Erskine et al., 2011) that a greater proportion of RGC axons projected ipsilaterally at the optic chiasm of constitutive *Nrp1* null mouse mutants compared with wild-type and heterozygous littermates (Fig. 1A), resulting in a significantly increased ipsilateral index (ratio of the fluorescence intensity in the ipsilateral and contralateral optic tracts; Fig. 1B; Fig. S1A). Additionally, the optic tracts split into two bundles each in a subset of *Nrp1*-null mutants (6/9) (Fig. 1A). The optic tracts of the mutants also appeared defasciculated and contained numerous axon exclusion zones (Fig. 1A, white arrowheads; Fig. 1C; Fig. S1B). NRP1 loss therefore causes several different defects in optic pathway organisation. Blood vessels bordering the optic pathway in *Nrp1* null mutants also were mispatterned (Fig. 1D). As observed previously in the hindbrain (Fantin et al., 2015; Gerhardt et al., 2004), neighbouring vessels in the diencephalon failed to interconnect and instead formed large, blind-ended tufts (Fig. 1D). Accordingly, our analyses of *Nrp1*-null mutants could not distinguish direct roles for NRP1 in RGCs from indirect effects of NRP1-dependent blood vessels on RGCs.

### *Brn3b^Cre^* drives efficient knockdown of *Nrp1* in RGCs

As attempts to ablate conditional *Nrp1* null (floxed) alleles in RGCs with *Six3-Cre* or Nes-Cre resulted in only partial NRP1 targeting (Fig. S2), we investigated whether a *Brn3b^Cre^* knock-in allele could selectively ablate NRP1 in RGCs without affecting blood vessel expression. *Brn3b* (*Pou4f2*) is expressed in RGCs during or shortly after their terminal cell division (Prasov and Glaser, 2012). For these experiments, we bred mice carrying two conditional *Nrp1* null alleles together with the *Rosa26^Yfp^* recombination reporter to *Nrp1^+/−^* mice carrying a *Brn3b^Cre^* knock-in allele (Simmons et al., 2016). The *Nrp1^+/−^* background was chosen, because this strategy maximises *Nrp1* knockdown as CRE-mediated *Nrp1* recombination is not 100% efficient, as shown previously in analogous experiments with other cell types (Fantin et al., 2013; Gu et al., 2003). In addition to leaving the RGC axon projection unaffected (Fig. 1A-C), the heterozygous NRP1 loss also does not cause obvious defects in blood vessel formation at the optic chiasm (Fig. 1D).

**Fig. 2.**
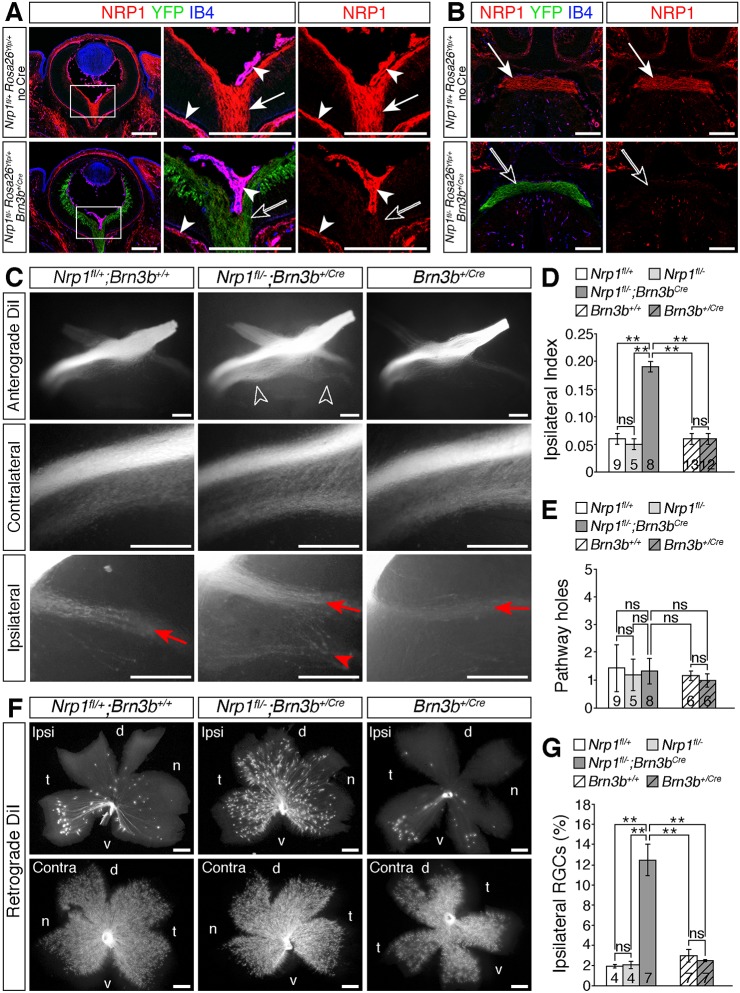
**NRP1 on RGC axons is required for midline crossing at the optic chiasm.** (A,B) Immunolabelling of E14.5 horizontal sections through the retina (A) and optic chiasm (B; anterior up) for NRP1 (red), IB4 (blue) and YFP (green) in control (*Nrp1^fl/+^;Rosa26^Yfp^^/+^*; no *Cre*) and RGC-specific NRP1 mutant (*Nrp1^fl/−^;Rosa26^Yfp/+^;Brn3b^+/Cre^*) embryos. Boxed regions in A are shown at higher magnification in the adjacent panels. Purple indicates co-labelling of NRP1 and IB4 in the overlay of all three channels; the single red (NRP1) channel is shown on the right. White arrows indicate NRP1-positive RGC axons, unfilled arrows loss of NRP1 from RGCs in the conditional mutants, white arrowheads NRP1-positive hyaloid and choroidal vessels. (C) Whole-mount views of RGC axons, labelled anterogradely with DiI in E14.5 control embryos (*Nrp1^fl/+^;Brn3b^+/+^*), mutants lacking *Nrp1* in RGCs (*Nrp1^fl/−^;Brn3b^+/Cre^*) or embryos carrying a *Brn3b^Cre^* allele, but no floxed gene (*Brn3b^+/Cre^*). Higher magnification views of the contralateral and ipsilateral optic tracts are shown beneath each panel. Unfilled arrowheads indicate the increased proportion of RGC axons projecting ipsilaterally in *Nrp1^fl/−^;Brn3b^+/Cre^* mutants, red arrows the normal position and organisation of the ipsilateral projection, red arrowhead the increased size and abnormal organisation of the ipsilateral projection in *Nrp1^fl/−^;Brn3b^+/Cre^* mutants. (D,E) Ipsilateral index (D) and total number of exclusion zones (holes) at the optic chiasm and in the contralateral optic tract (E) in E14.5 *Nrp1^fl/−^;Brn3b^+/Cre^* and *Brn3b^+/Cre^* embryos and littermate controls. (F,G) Flat-mounted ipsilateral (ipsi) and contralateral (contra) retinas (F) and proportion of ipsilateral RGCs relative to total number of RGCs in both eyes (G) after retrograde labelling from one optic tract in E15.5 *Nrp1^fl/−^;Brn3b^+/Cre^* and *Brn3b^+/Cre^* embryos and littermate controls. d, dorsal, n, nasal, t, temporal, v, ventral. Data in D,E,G are shown as mean±s.e.m.; **P<0.01; ns, not significant (D,E: one-way ANOVA with post-hoc Tukey; G: Kruskal–Wallis rank sum test with post-hoc Tukey). Numbers on bars indicate the number of embryos analysed for each genotype. Scale bars: 200 µm (A,B); 250 µm (C,F).

The *Brn3b^Cre^* allele efficiently activated *Rosa26^Yfp^* expression in RGCs (arrows in both Fig. 2A,B and Fig. S3), but spared endothelial cells in and around the eye and at the optic chiasm (arrowheads in both Fig. 2A,B and Fig. S3). In agreement, immunolabelling with an antibody specific for NRP1 (Fantin et al., 2010) demonstrated that endothelial NRP1 expression in the hyaloid, choroidal and diencephalic vasculature was maintained, although at slightly lower levels than in wild types due to the presence of the heterozygous *Nrp1*-null allele (arrowheads in both Fig. 2A,B and Fig. S3). In contrast, immunolabelling confirmed effective NRP1 knockdown in RGC axons of *Nrp1^fl/−^;Brn3b^+/Cre^* mice, both in the eye and at the optic chiasm (unfilled arrows in Fig. 2A,B and in Fig. S3). These findings demonstrate that *Brn3b^Cre^* is a suitable tool to ablate NRP1 in RGCs.

### RGCs express NRP1 to promote axon crossing at the optic chiasm and axon fasciculation in the optic tract

Anterograde DiI labelling at E14.5 revealed that ablating NRP1 from RGCs phenocopied the midline-crossing defects of full *Nrp1* null mutants (Fig. 2C). Thus, in 8/8 *Nrp1^fl/−^;Brn3b^+/Cre^* mutants, the size of the ipsilateral projection was substantially larger than in control mice (Fig. 2C), reflected in a significantly increased ipsilateral index (Fig. 2D), similar to full *Nrp1* mutants (Fig. 1A). Anterograde DiI labelling of E14.5 *Brn3b^+/Cre^* mutants excluded the possibility that the insertion of *Cre* into the *Brn3b* locus had compromised RGC axon guidance (Fig. 2C,D).

Retrograde DiI labelling from one optic tract at E15.5, followed by flat-mounting the ipsilateral and contralateral retinas, confirmed that an increased proportion of RGCs had projected ipsilaterally in *Nrp1^fl/−^;Brn3b^+/Cre^* mutants compared with littermate controls and *Brn3b^+/Cre^* mice (Fig. 2F,G). In the wild-type mouse retina, ipsilaterally projecting RGCs originate predominately in the ventral temporal crescent, the site of binocular overlap in the visual field (Erskine and Herrera, 2014) (Fig. 2F). However, retrogradely labelled RGCs were distributed throughout the ipsilateral retina in *Nrp1^fl/−^;Brn3b^+/Cre^* mutants (Fig. 2F). This phenotype cannot be explained by excessive specification of ipsilateral RGCs, as specification proceeds normally in full *Nrp1* null mutants (Erskine et al., 2011). The significant increase in ipsilaterally projecting RGCs in *Nrp1^fl/−^;Brn3b^+/Cre^* mutants therefore reflects a failure of presumptive contralateral RGC axons to cross the chiasm midline.

In addition to the midline-crossing defects, anterograde DiI labelling revealed defasciculation of the ipsilateral optic tract in *Nrp1^fl/−^;Brn3b^+/Cre^* mutants (Fig. 2C), as observed in full *Nrp1* null mutants (Fig. 1A). In marked contrast to full *Nrp1* mutants, the incidence of exclusion zones in the optic tracts was not significantly different in *Nrp1^fl/−^;Brn3b^+/Cre^* mutants and controls (Fig. 2E). We therefore investigated whether endothelial NRP1 expression also contributes to optic pathway organisation.

### Endothelial NRP1 is required for RGC axon organisation in the optic tract

**Fig. 3.**
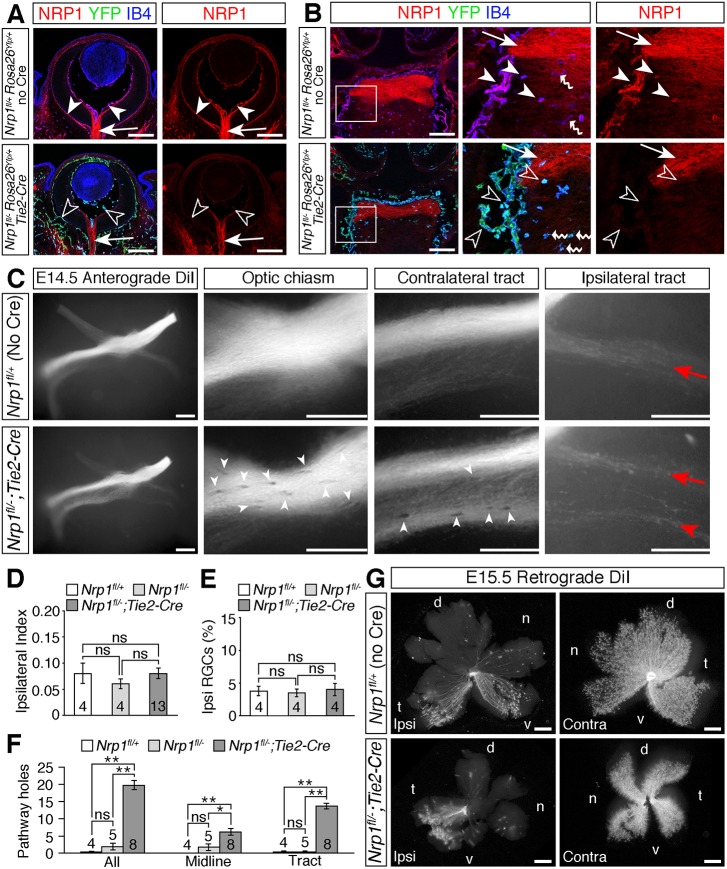
**Loss of NRP1 from endothelial cells impairs RGC axon organisation.** (A,B) Immunolabelling of E14.5 horizontal sections through the retina (A) and optic chiasm (B; anterior up) for NRP1 (red), IB4 (blue) and YFP (green) in control embryos (*Nrp1^fl/+^;Rosa26^Yfp/+^*; no Cre) and endothelial *Nrp1* mutant embryos (*Nrp1^fl/−^;Rosa26^Yfp/+^;Tie2-Cre*). All three channels together or the single red (NRP1) channel are shown, as indicated. Purple indicates colocalisation of NRP1 and IB4 in endothelial cells and microglia. Boxed regions in B are shown at higher magnification in the adjacent panels. White arrowheads indicate NRP1-positive endothelial cells, unfilled arrowheads loss of NRP1 from endothelial cells in mutants, white arrows NRP1-positive RGC axons, wavy arrows IB4-positive microglia. (C) Whole-mount views of RGC axon bundles, labelled anterogradely with DiI in E14.5 control embryos (*Nrp1^fl/+^*, but no Cre) or littermates lacking NRP1 in endothelial cells (*Nrp1^fl/−^;Tie2-Cre*). Higher magnification views of the optic chiasm, contralateral optic tract and ipsilateral optic tract are shown to the right. Red arrows indicate the normal position and organisation of the ipsilateral projection, red arrowhead the splitting of the ipsilateral projection in the mutant, white arrowheads exclusion zones in the RGC axon bundles. (D-F) Ipsilateral index at E14.5 (D), proportion of ipsilateral RGCs relative to total number of RGCs in both eyes at E15.5 (E) and total number of exclusion zones (holes) in the optic pathway, at the optic chiasm (midline) or in the contralateral optic tract at E14.5 (F). Data are shown as mean±s.e.m.*P<0.05, **P<0.01; ns, not significant (D,E: one-way ANOVA; F: Kruskal–Wallis rank sum test with post-hoc Tukey). Numbers on bars indicate number of embryos analysed for each genotype. (G) Flat-mounted ipsilateral (ipsi) and contralateral (contra) retinas after retrograde labelling of RGC axons from one optic tract in E15.5 control embryos or mutant littermates lacking *Nrp1* in endothelial cells. Although the retinal area is smaller in *Nrp1^fl/−^;Tie2-Cre* mutants, the ipsilaterally projecting RGCs are restricted to the ventrotemporal crescent, similar to the control retina. d, dorsal, n, nasal, t, temporal, v, ventral. Scale bars: 200 µm (A,B); 250 µm (C,G).

To generate endothelial *Nrp1* null mice, we bred mice carrying the endothelial *Tie2-Cre* transgene to *Nrp1^+/−^* mice and then bred the resulting *Nrp1^+/−^;Tie2-Cre* mice to *Nrp1^fl/fl^* mice carrying the *Rosa26^Yfp^* recombination reporter (Fantin et al., 2013). *Tie2-Cre* drives recombination in blood vessels throughout the embryo (Kisanuki et al., 2001), including the eye and diencephalon (Fig. S4, arrowheads). Double labelling for YFP and NRP1 confirmed that *Nrp1* deletion in endothelial cells of the eye and around the optic chiasm was successful in *Nrp1^fl/^;Tie2-Cre* mutants, whilst NRP1 expression in RGCs was maintained, albeit at slightly lower levels due to the *Nrp1^+/−^* allele (Fig. 3A,B; Fig. S4).

Anterograde DiI labelling at E14.5 demonstrated normal RGC axon crossing at the optic chiasm in *Nrp1^fl/−^;Tie2-Cre* mutants lacking endothelial NRP1 (Fig. 3C,D). Probably owing to defective choroidal vascularisation (Marneros et al., 2005), the retinal area was substantially decreased in *Nrp1^fl/−^;Tie2-Cre* mutants (Fig. 3G). Nevertheless, retrograde DiI labelling from the optic tract at E15.5 confirmed that a normal proportion of RGCs projected ipsilaterally in these mice (Fig. 3E,G), and the restricted origin of ipsilaterally projecting RGCs to the ventral-temporal crescent of the retina was also maintained (Fig. 3G). These experiments established that midline sorting of RGC axons was not defective in endothelial *Nrp1* mutants.

Although midline crossing occurred normally, optic tract organisation was perturbed in endothelial *Nrp1* null mutants. In 6/13 (46%) of *Nrp1^fl/−^;Tie2-Cre* mutants, but not in control embryos, the ipsilateral optic tract had split into two distinct bundles (Fig. 3C). Additionally, RGC axon bundles had numerous exclusion areas both at the midline and in the optic tract in 8/8 endothelial *Nrp1* mutants examined (Fig. 3C,F, white arrowheads). These findings demonstrate that loss of NRP1 from blood vessels has no impact on midline crossing at the optic chiasm, but does affect optic tract morphology.

### Endothelial NRP1 restricts vessel sprouting at the diencephalic midline

**Fig. 4.**
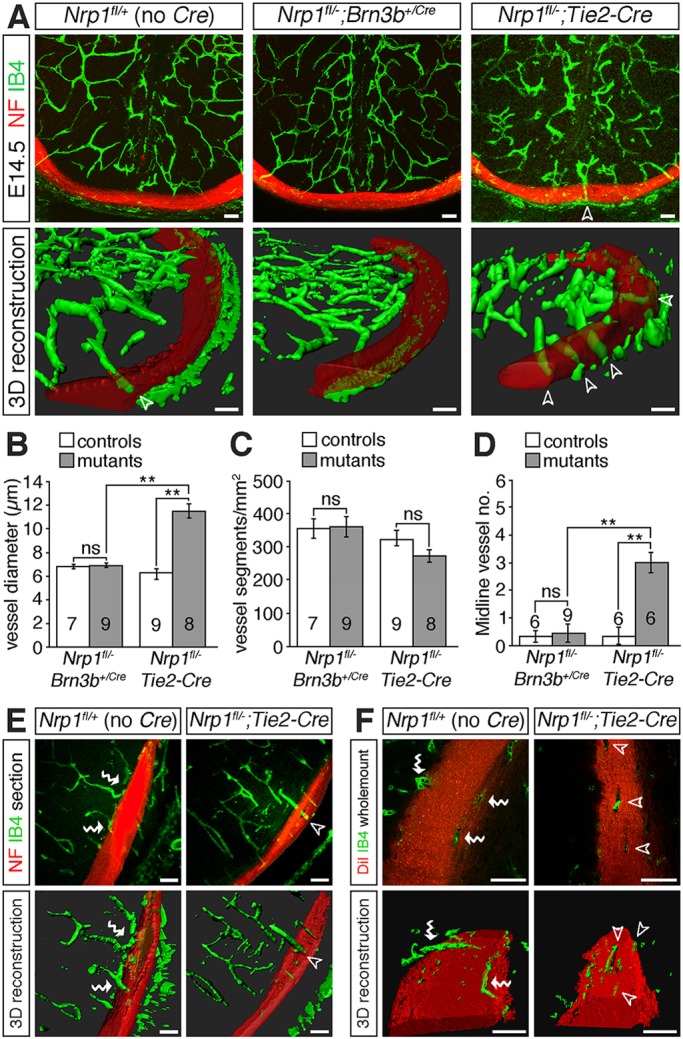
**Endothelial NRP1 loss impairs nerve-blood vessel co-patterning.** (A) Confocal *z*-stacks (top panels) and 3D reconstructions of *z*-stacks (bottom panels) of coronal sections through the E14.5 diencephalon of control embryos (*Nrp1^fl/+^*, no Cre), mutants lacking *Nrp1* in RGCs (*Nrp1^fl/−^;Brn3b^+/Cre^*) or mutants lacking NRP1 in endothelial cells (*Nrp1^fl/−^;Tie2-Cre*). Blood vessels were labelled with IB4 (green) and nerves with antibodies against neurofilaments (NF; red). (B-D) Mean±s.e.m. vessel diameter (B), vessel density (C) and number of midline vessel sprouts (D) in control littermates and mutants lacking *Nrp1* in RGCs (*Nrp1^fl/−^;Brn3b^+/Cre^*) or endothelial cells (*Nrp1^fl/−^;Tie2-Cre*). **P<0.01; ns, not significant (one-way ANOVA with post-hoc Tukey). Numbers on bars indicate the number of embryos analysed for each genotype. (E,F) Confocal *z*-stacks and 3D reconstructions of *z*-stacks of optic tracts from E14.5 control embryos and mutants lacking *Nrp1* in endothelial cells, viewed in coronal sections (E) or as whole-mounts (F). Samples were labelled with antibodies against neurofilaments (E, red) or DiI (F, red) to label the nerves and IB4 (green) to label blood vessels. The red channel has been made semi-transparent in the 3D reconstructions in A,E,F. Unfilled arrowheads indicate vessel sprouts extending through RGC axon bundles, white wavy arrows vessels growing along the surface of the RGC axon bundles. Scale bars: 50 µm.

To compare the relationship between RGC axons and blood vessels in *Nrp1^fl/−^;Tie2-Cre* and *Nrp1^fl/−^;Brn3b^+/Cre^* mutants, we combined endothelial IB4 staining with RGC axon labelling (Fig. 4). Vessel patterning was indistinguishable in the diencephalon of E14.5 *Nrp1^fl/−^;Brn3b^+/Cre^* mutants and control littermates (Fig. 4A-D), correlating with the observation that these mutants had few optic pathway holes (Fig. 2C,E). In agreement with the prior finding that *Nrp1^fl/−^;Tie2-Cre* mice have less severe vascular patterning defects than full *Nrp1* null mice, overall vessel density was not significantly different in *Nrp1^fl/−^;Tie2-Cre* mutants compared with control littermates (Fig. 4C). The reduced severity is explained by the selective retention of NRP1 in a subset of recombination-resistant endothelial cells that partially rescue vessel sprouting (Fantin et al., 2013). Nevertheless, the diameter of vessels was increased significantly in E14.5 *Nrp1^fl/−^;Tie2-Cre* mutants compared with control littermates (Fig. 4A,B), and vessel patterning at the diencephalic midline was also perturbed, with a significant increase in the number of vessels extending perpendicularly through the axon bundles at the optic chiasm (Fig. 4A,D, unfilled arrowheads).

The spatial relationship of blood vessels and RGC axons was also altered in the optic tracts of *Nrp1^fl/−^;Tie2-Cre* mutants. In control brains, most vessels were positioned around or along the surface of the optic tracts, but rarely within the axon bundles (Fig. 4E,F, wavy arrows). Moreover, the few vessels that penetrated the axon bundles were usually located at the edge of the bundles and followed an oblique, rather than perpendicular course (Fig. 4F). In contrast, vessel sprouts extended perpendicularly through the optic tracts of *Nrp1^fl/−^;Tie2-Cre* mutants in the areas where holes were present in the axon bundles (Fig. 4E,F, unfilled arrowheads). These observations demonstrate that axon exclusion zones in the diencephalon of endothelial *Nrp1* null mutants occur in areas harbouring abnormal blood vessels.

### Aberrant vessel patterning underlies the emergence of optic pathway holes

**Fig. 5.**
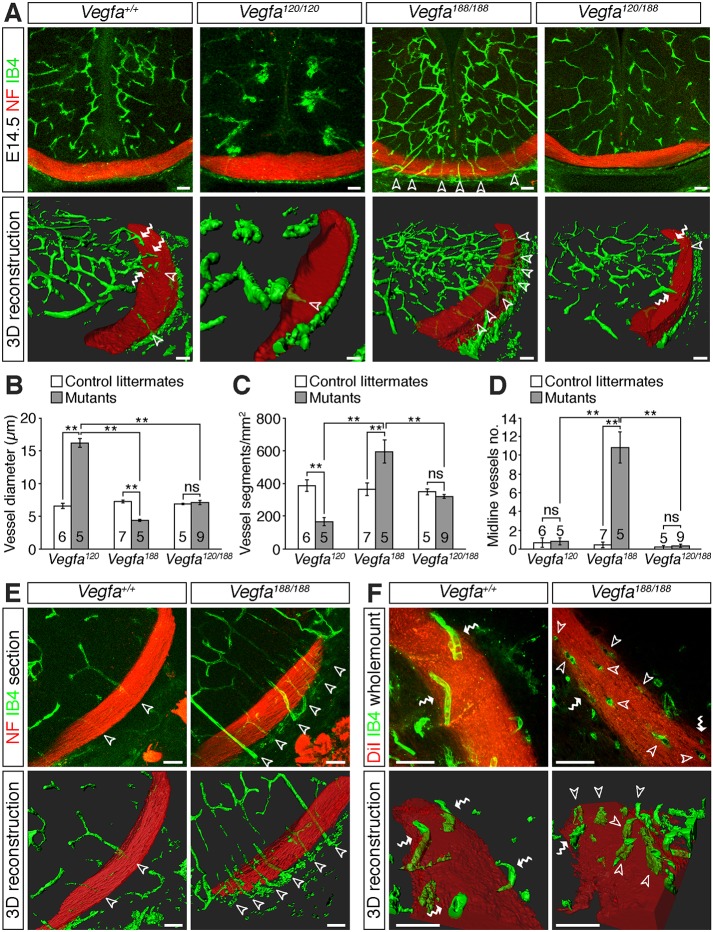
**Vascular patterning defects in the diencephalon of *Vegfa* isoform mutants.** (A) Confocal *z*-stacks (top panels) and 3D reconstructions of *z*-stacks (bottom panels) of coronal sections through the diencephalon of E14.5 control embryos and mutants expressing specific *Vegfa* isoforms. Blood vessels were labelled with IB4 (green) and nerves with antibodies against neurofilaments (NF; red). (B-D) Vessel diameter (B), vessel density (C) and number of midline vessel sprouts (D) in mutants expressing specific *Vegfa* isoforms and control littermates (mean±s.e.m.). **P<0.01; ns, not significant (one-way ANOVA with post-hoc Tukey). Numbers on bars indicate the number of embryos analysed for each genotype. (E,F) Confocal *z*-stacks (top images) and 3D reconstructions of *z*-stacks (bottom images) of optic tracts from E14.5 control embryos and *Vegfa^188/188^* mutants, shown in coronal sections (E) or as whole-mounts (F). Samples were labelled with antibodies against neurofilaments (E, red) or DiI (F, red) to label the nerves and IB4 (green) to label blood vessels. The red channel has been made semi-transparent in the 3D reconstructions in A,E,F. Unfilled arrowheads indicate vessel sprouts extending through the RGC axon bundles, white wavy arrows vessels growing along the surface of the RGC axon bundles. Scale bars: 50 µm.

To determine whether the emergence of optic pathway holes is specific to mutants lacking NRP1 in endothelial cells or a general consequence of defective brain vascularisation, we analysed the patterning of RGC axons and blood vessels bordering the optic pathway in two additional types of mutants with defective vascular patterning. First, we examined *Vegfa^120/120^* mutants, which express VEGF_120_, but lack VEGF_164_ and VEGF_188_ (Carmeliet et al., 1999) and therefore establish defective VEGF-A gradients for vascular morphogenesis (Ruhrberg et al., 2002). As reported for the hindbrain (Ruhrberg et al., 2002) and retina (Stalmans et al., 2002), the diencephalon of *Vegfa^120/120^* mutants contained vessels that were wider and branched less frequently than in wild-type littermates; accordingly, vascular density was significantly reduced in these mutants (Fig. 5A-C). Second, we examined *Vegfa^188/188^* mutants that express VEGF_188_ at the expense of the other VEGF-A isoforms (Stalmans et al., 2002). In contrast to *Vegfa^120/120^* mutants, *Vegfa^188/188^* mutants had abnormally thin and hyperbranched vessels in the diencephalon (Fig. 5A-C), as has been reported for the hindbrain (Ruhrberg et al., 2002) and retina (Stalmans et al., 2002). The vascular patterning defects in these mice are distinct from those of *Nrp1* null mutants (compare Fig. 1D and Fig. 5A) because NRP1 does not promote brain vascularisation as a VEGF-A receptor (Fantin et al., 2014; Gelfand et al., 2014). Consequently, *Vegfa* mutant mice provide complementary models for studying the impact of vascular patterning defects on optic pathway formation.

**Fig. 6.**
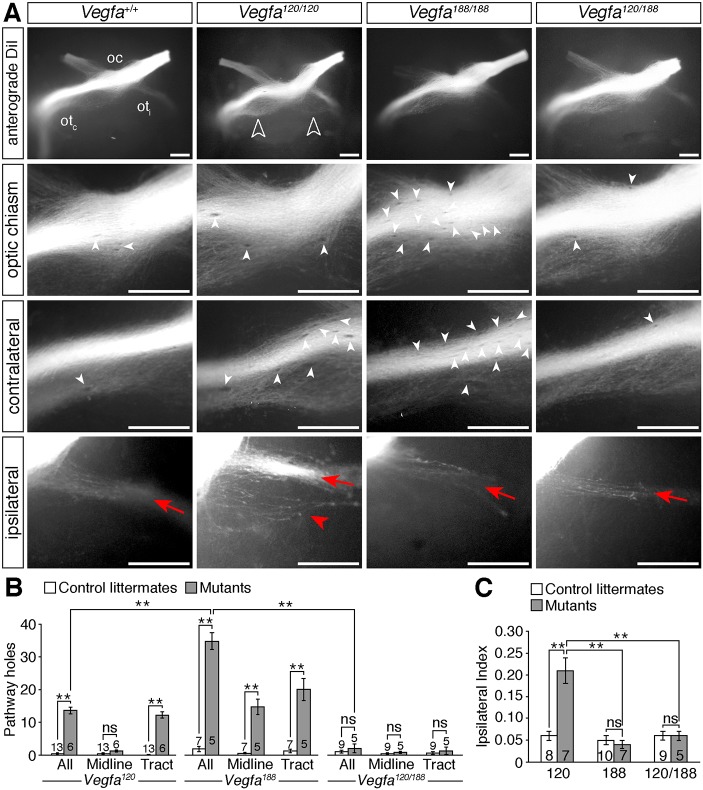
**Optic pathway defects in *Vegfa* isoform mutants.** (A) Whole-mount views of RGC axons, labelled anterogradely with DiI in E14.5 control embryos or mutants expressing specific *Vegfa* isoforms. Higher magnification views of the optic chiasm and contralateral and ipsilateral optic tracts are also shown. on, optic nerve; ot_c_, contralateral optic tract; ot_i_, ipsilateral optic tract. Unfilled arrowheads indicate the increased ipsilateral projection in *Vegfa^120/120^* mutants, red arrows the normal position and organisation of the ipsilateral projection, red arrowhead the defasciculation and splitting of the ipsilateral projection in *Vegfa^120/120^* mutants, white arrowheads exclusion zones in the RGC axon bundles. (B,C) Total number of optic pathway exclusion zones (holes) and at the optic chiasm (midline) and in the contralateral optic tract (B) and ipsilateral index (C) in E14.5 control embryos and mutant littermates. Data are shown as mean±s.e.m. **P<0.01; ns, not significant (one-way ANOVA with post-hoc Tukey). Numbers on bars indicate number of embryos analysed for each genotype. Scale bars: 250 µm.

In addition to the opposing defects in vessel diameter and branching frequency throughout the *Vegfa^120/120^* and *Vegfa^188/188^* diencephalon, the diencephalic midline remained relatively avascular in *Vegfa^120/120^* mutants, whereas *Vegfa^188/188^* mutants had numerous ectopic vessels extending perpendicularly through the RGC axon bundles (Fig. 5A,D, unfilled arrowheads). Correlating with these differences in vascular patterning at the midline, anterograde DiI labelling demonstrated a significant increase in the number of optic pathway holes at the optic chiasm of E14.5 *Vegfa^188/188^*, but not *Vegfa^120/120^*, mutants (Fig. 6A,B, white arrowheads). Within the optic tracts, *Vegfa^120/120^* mutants had significantly more holes than control littermates, but fewer than *Vegfa^188/188^* mutants (Fig. 6A,B). The position of the optic tract holes in *Vegfa^188/188^* mutants correlated with the location of vessels passing perpendicularly through the axon bundles (Fig. 5E,F, unfilled arrowheads).

Whereas *Vegfa^120/120^* mutants displayed an increase in ipsilaterally projecting RGC axons at the optic chiasm, *Vegfa^188/188^* mutants did not (Fig. 6A,C), in agreement with our previous finding that VEGF_188_ can bind NRP1 and is sufficient for contralateral axon growth in the absence of VEGF_164_ (Tillo et al., 2015). Compound *Vegfa^120/188^* mice, which express VEGF_120_ and VEGF_188_, also lacked midline projection defects (Fig. 6A,C) (Tillo et al., 2015). In contrast to *Vegfa^188/188^* mutants, *Vegfa^120/188^* mutants did not have obvious vascular patterning defects in the diencephalon (Fig. 5A-D) and correspondingly have a similar number of optic pathway holes at the midline and in the optic tracts as controls (Fig. 6A,B). These observations demonstrate that defective vascular patterning impacts on optic pathway morphology, and that ectopic vessel sprouts and increased vessel diameter are associated with the emergence of axon exclusion zones, and occur independently of axon-sorting defects at the midline.

### Ectopic vessel sprouts are present prior to the ingrowth of RGC axons

**Fig. 7.**
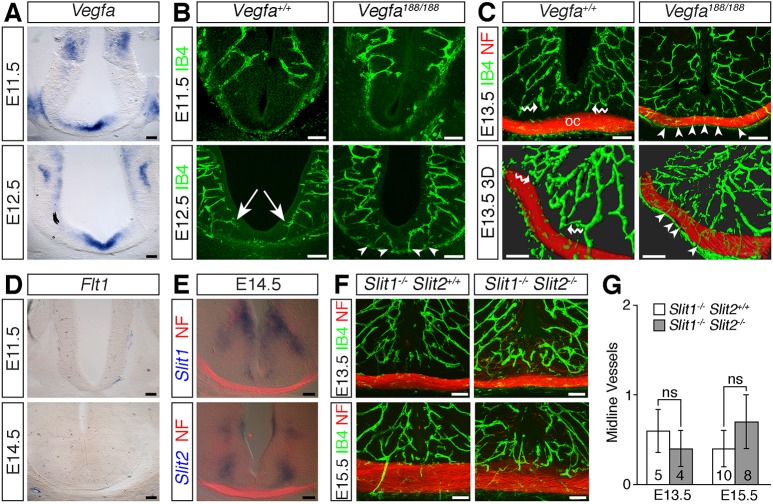
**Ectopic vessels sprout at the *Vegfa^188/188^* midline prior to axon ingression.** (A) *Vegfa in situ* hybridisation on coronal sections of E11.5 and E12.5 diencephalon. (B,C) Confocal *z*-stacks (B) and 3D reconstruction of confocal *z*-stacks (C) of coronal sections through wild-type and *Vegfa^188/188^* optic chiasm (oc), labelled with IB4 to visualise blood vessels (green) and antibodies against neurofilaments (NF) to visualise nerves (red). White arrows indicate medially extending vessel in the subventricular plexus, white arrowheads midline vessel sprouts, white wavy arrows vessels growing around RGC axon bundles. (D,E) *In situ* hybridisation for *Flt1*, *Slit1* or *Slit2* on coronal sections through E11.5 and E14.5 diencephalon; sections in E have been double-labelled with antibodies against neurofilament (red). (F) Confocal *z*-stacks of coronal sections through the optic chiasm of *Slit1^−/−^* and *Slit1^−/−^;Slit2^−/−^* embryos, labelled with IB4 to visualise blood vessels and antibodies against neurofilaments to visualise nerves. (G) Midline vessel sprouts in slit mutants (mean±s.e.m.). Numbers on bars indicate the number of embryos of each age and genotype analysed; ns, not significant (Student's two-tailed unpaired *t*-test). Scale bars: 100 µm.

To understand the relationship of growing axons and vessels at the chiasm midline, we first determined the spatiotemporal course of angiogenesis in the developing diencephalon of wild-type mice. At E11.5, vessel sprouts have begun to extend from the perineural vascular plexus towards the ventricle, but, despite strong *Vegfa* expression at the ventral midline (Fig. 7A), the ventral region of the diencephalic anlage remained devoid of vessels (Fig. 7B). By E12.5, vessel sprouts have begun to ingress into more ventral regions of the diencephalon, but only a few perpendicular vessel sprouts were visible adjacent to the midline; instead, the subventricular vascular plexus in the midline area appeared to arise predominately from medial branches of laterally positioned vessels (Fig. 7B, arrows). Consequently, as the first RGC axons extended through the ventral midline between E12.5 and E13.5, they grew through a region with few vessels. At E13.5, as the vessel network continued to expand and remodel, vessel sprouts from the subventricular plexus in the midline region extended ventrally towards the perineural vascular plexus, but most vessels grew around, rather than through, the RGC axon bundles (Fig. 7C, wavy arrows). The optic chiasm in wild-type embryos therefore forms in a region of the diencephalon that is largely avascular.

We next investigated how defective vessel patterning relates to the emergence of axon exclusion zones in *Vegfa^188/188^* mutants. Whereas the chiasm midline was largely avascular in E12.5 wild types, perpendicular vessel sprouts were present at the ventral midline of *Vegfa^188/188^* mutant littermates, prior to the ingrowth of RGC axons (Fig. 7B, arrowheads). Moreover, the number of such vessels sprouts increased as development proceeded (Fig. 7C, arrowheads). Accordingly, to progress through the optic chiasm, the ingrowing RGC axons in *Vegfa^188/188^* mutants would have needed to navigate around ectopic vessels positioned along their path.

We next considered whether known repulsive molecules prevent vascular ingression at the chiasmatic midline. Soluble FLT1 acts as an inhibitory decoy receptor for VEGF-A and regulates the balance of pro-angiogenic VEGF-A signalling to control vessel sprouting in the spinal cord (Himmels et al., 2017; Wild et al., 2017). However, *in situ* hybridisation did not detect *Flt1* expression in a pattern consistent with preventing vessel sprouting into the murine diencephalic midline (Fig. 7D). Slits are alternative candidates for midline repulsion of vessels, because they can inhibit endothelial cell migration in the presence or absence of VEGF-A *in vitro* (Jones et al., 2008; Park et al., 2003) and because *Slit1* and *Slit2* (but not *Slit3*) are expressed dorsally to the developing optic chiasm (Erskine et al., 2000) (Fig. 7E). However, endothelial labelling of coronal sections through the optic chiasm of *Slit1^−/−^* single or *Slit1^−/−^;Slit2^−/−^* compound mutants demonstrated that the diencephalon midline remained vessel free in the absence of slits (Fig. 7F,G). Further work will therefore be required to establish whether inhibitory signalling by unidentified chiasmatic regulatory cues is required to prevent vessel sprouting at the diencephalic midline.

### Growing blood vessels do not repel RGC axons *in vitro* or *in vivo*

**Fig. 8.**
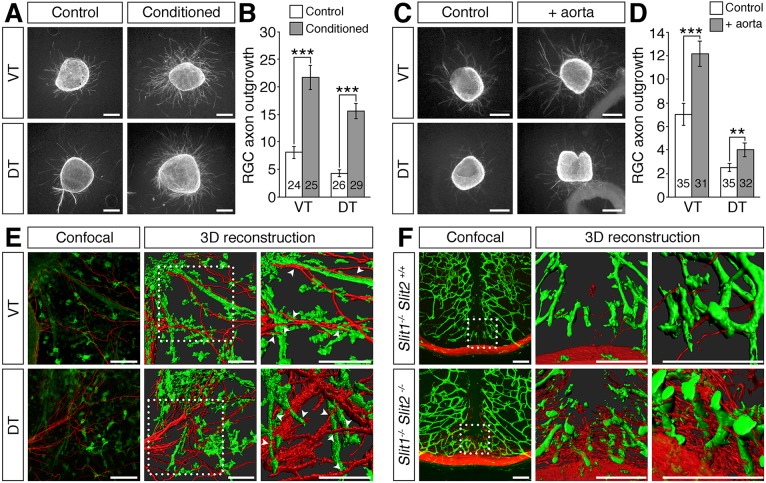
**Vessels are not inhibitory to RGC axon growth *in vitro* or *in vivo*.** (A-F) Retinal explants from E14.5 wild-type ventrotemporal (VT; source ipsilateral RGCs) or dorsotemporal (DT; source contralateral RGCs) retina cultured for 48 h in collagen gels with control or endothelial cell-conditioned medium (A,B) or co-cultured with an aortic ring (C,D). Cultures were stained for β-tubulin (A,C) and axon outgrowth quantified (B,D). Data are the mean±s.e.m. of at least three independent experiments. The number of explants analysed for each condition is indicated on each bar. ***P<0.001, **P<0.01 (Student's two-tailed unpaired *t*-test). In E, blood vessels were labelled with IB4 (green) and nerves with antibodies against neurofilaments (NF; red) and are shown as confocal *z*-stacks (left) and 3D reconstructions of the *z*-stacks (centre). The boxed regions are shown at higher magnification (right). Arrowheads indicate axons growing across or along the surface of vessels. (F) Confocal *z*-stacks and 3D reconstructions of the boxed region in the *z*-stacks at different magnifications of coronal sections through the diencephalon of E13.5 slit mutants. Blood vessels were visualised with IB4 (green) and nerves with antibodies against neurofilaments (red). Scale bars: 250 µm (A,C); 100 µm (E,F).

Axon exclusion zones in mice with abnormal midline vascularisation might develop through active repulsion of RGC axons by vessels located along their path. To test whether endothelial cells secrete factors that are inhibitory to RGC axon outgrowth, we cultured retinal explants in control or endothelial cell-conditioned medium and quantified the extent of retinal axon outgrowth. We found that retinal axon outgrowth was significantly greater from explants cultured in endothelial cell-conditioned medium than control medium (Fig. 8A,B). Similarly, co-culturing retinal explants with aortic rings significantly increased axon outgrowth (Fig. 8C,D; Fig. S5). These observations suggest that endothelial cells secrete factors that in sum have a growth-promoting effect on retinal axon outgrowth. In addition, we found no evidence for contact-dependent repulsion of retinal axons by endothelial cells in co-cultures of retinal explants with aortic rings. Thus, retinal axons grew amongst the vessels extending from the aortic rings, with some axons growing along and across the surface of the vessels (Fig. 8E, arrowheads).

We also examined how RGC axons are affected by blood vessels when they stray from their normal, poorly vascularised path into more densely vascularised diencephalic areas. For these experiments, we took advantage of *Slit1^−/−^* single and *Slit1^−/−^;Slit2^−/−^* compound mutants, because *Slit1* and *Slit2* expression is not detectable in endothelial cells (Fig. 7E) (Erskine et al., 2000; Rama et al., 2015), and loss of slit genes causes some RGC axons to misproject into dorsal regions of the diencephalon (Plump et al., 2002). Consistent with prior findings, a few RGC axons in *Slit1^−/−^* single mutants strayed from the chiasm into more dorsal regions of the diencephalon, where they grew amongst vessels (Fig. 8F). In *Slit1^−/−^;Slit2^−/−^* compound mutants, many more RGC axons strayed dorsally from the chiasm and intermingled with blood vessels, and these ectopic axons grew around and across the surface of vessels (Fig. 8F). Together, these data demonstrate that endothelial cells *in vitro* and blood vessels *in vivo* do not actively repel RGC axons. It is therefore likely that large or excessively dense ectopic blood vessels positioned along the RGC axon path provide a physical barrier that forces axons to navigate around them, creating exclusion zones that appear as holes in the axon bundles.

## DISCUSSION

NRP1 plays an important role in the pre-target sorting of axons in the mammalian olfactory system and corpus callosum (Imai et al., 2009; Zhou et al., 2013). Using a *Brn3b^Cre^* knock-in allele, we have now provided *in vivo* evidence for a cell-autonomous requirement of NRP1 signalling in RGCs to regulate midline crossing of their axons at the optic chiasm as well as axon fasciculation, particularly in the ipsilateral optic tract. The role of NRP1 in midline crossing was consistent with prior evidence that contralaterally specified RGCs express NRP1 *in situ* and that VEGF-A stimulates the growth of their axons *in vitro* (Erskine et al., 2011). Although NRP1 was characterised initially as a cell adhesion receptor in the central nervous system (Takagi et al., 1995), its precise role in axon fasciculation has been studied mainly in the peripheral nervous system, where semaphorin-induced NRP1-mediated axon-axon interactions regulate the fasciculation of sensory and motor axons (Huettl et al., 2011). However, semaphorin signalling through NRP1 is not required for axon fasciculation in the optic tract (Erskine et al., 2011; Hörnberg et al., 2016). NRP1 expression by contralateral RGC axons clearly promotes the fasciculation of these commissural axons, but whether this occurs through axon-axon interactions induced by other NRP1 ligands (such as VEGF-A) or ligand-independent NRP1 interactions remains to be established.

In contrast to NRP1 expression by contralaterally projecting axons, ipsilateral RGCs do not express NRP1, and ipsilateral optic tract defasciculation after *Brn3b^Cre^*-mediated NRP1 deletion was therefore surprising (Erskine et al., 2011). Lack of NRP1 expression in ipsilateral RGCs makes it unlikely that NRP1 directly controls the fasciculation of their axons. A more plausible explanation might be that misrouted ipsilaterally projecting axons (from contralaterally specified RGC neurons) fail to integrate properly with the normal ipsilaterally projecting axons (from ipsilaterally specified RGC neurons), and that this failed integration disrupts ipsilateral tract morphology. In agreement with this idea, axons from ipsilaterally specified RGC neurons normally occupy a relatively restricted region within the optic tract that is distinct from that occupied by contralaterally projecting axons (Colello and Coleman, 1997; Soares and Mason, 2015). Moreover, the ipsilateral optic tracts of all known types of mutants with excessive ipsilateral projections appear defasciculated (e.g. Erskine et al., 2011; Kuwajima et al., 2012; Pinter and Hindges, 2010).

In addition to an increased ipsilateral projection and excessive defasciculation, the optic tracts contained numerous holes in *Nrp1* mutants owing to the exclusion of axons from areas that were populated by ectopic and/or abnormal vessels. The presence of these axon exclusion zones could not be explained by loss of NRP1 from RGC axons, as they were absent from the optic tracts of RGC-selective *Nrp1* null mutants. Instead, we observed similar exclusion zones in the optic tracts of endothelial *Nrp1* mutants, pointing to vascular patterning defects as the cause. In agreement, optic tract holes were also present in two other types of mutants with defective blood vessel patterning, *Vegfa^120/120^* and *Vegfa^188/188^* mice. Thus, both types of *Vegfa* mutants, similar to endothelial *Nrp1* mutants, had axon exclusion zones precisely in the areas where ectopic or abnormal vessels were present.

We considered the possibility that axon exclusion zones in endothelial NRP1 and VEGF-A isoform mutants were caused by the release of repulsive signals from ectopic blood vessels that were positioned in the path of growing RGC axons. However, our experiments did not provide evidence that blood vessels secrete factors or have cell surface properties that are inhibitory to RGC axon outgrowth; instead, blood vessels appeared to release factors that promoted RGC axon growth. The simplest explanation for axonal exclusion zones in mice with vascular brain defects might therefore be that abnormal vessels present a physical obstacle to RGC axon growth. Thus, our data are most compatible with the idea that the co-patterning of nerves and vessels in the optic pathway is not an active process driven by reciprocal signalling, but reflects passive co-existence, made possible by the small diameter and specific distribution of vessels in the diencephalon, combined with the ability of axons to activate mechanisms that enable them to navigate around vessels. As RGC axons appear to follow a relatively normal path after they have navigated around any aberrant vessels, the impact of the axon exclusion zones in *Nrp1* and *Vegfa* mutants on visual function is likely to be minor.

Our spatiotemporal analysis of vascular ingression at the diencephalic midline demonstrated vessel exclusion at a time when axons readily progress through this region. This observation was unexpected, as the midline area expresses high *Vegfa* levels that promote contralateral RGC axon crossing (Erskine et al., 2011). Accordingly, it is likely that the midline region contains anti-angiogenic cues that counteract the pro-angiogenic function of VEGF-A without compromising VEGF-A signalling in RGCs to stimulate axon growth. This balance of pro-angiogenic and anti-angiogenic cues appears to be perturbed in *Vegfa^188/188^* mutants. VEGF_188_ levels are higher in *Vegfa^188/188^* mutants relative to wild-type mice, because the genetic targeting strategy employed to generate these mutants abolishes alternative splicing of the *Vegfa* gene without compromising overall VEGF-A levels (Stalmans et al., 2002). VEGF_188_ is normally sequestered in the extracellular matrix and at the surface of secreting cells, whereas VEGF_120_ is highly diffusible and VEGF_164_ has intermediate properties (Park et al., 1993; Ruhrberg et al., 2002). Increased local VEGF-A retention at the diencephalic midline of *Vegfa^188/188^* mutants might therefore underlie increased vessel ingression compared with wild type, in which this tissue area expresses mostly VEGF_120_ and VEGF_164_, but only low levels of VEGF_188_ (Tillo et al., 2015). In agreement with the idea that high levels of extracellular matrix-retained VEGF-A promote vessel ingression into normally avascular midline areas, excessive sprouting into the midline region has been observed previously in the hindbrain of *Vegfa^188/188^* embryos (Ruhrberg et al., 2002). Moreover, the exogenous expression of VEGF_189_, the human equivalent of VEGF_188_, induces additional ingression points into the avian neural tube (James et al., 2009).

Our finding that the chiasmatic midline area is normally avascular agrees with prior work which showed that vessels in the spinal cord normally flank, but do not grow across the ventral midline, despite the abundant contralateral projection of axons (James et al., 2009; Kurz et al., 1996; Nakao et al., 1988). The question therefore arises whether inhibitory signals normally outweigh attractive signals for vessels at the CNS midline. The balance of soluble FLT1 and VEGF-A levels regulates initial vessel sprouting into the zebrafish spinal cord (Wild et al., 2017). However, we found that *Flt1* is not expressed in the ventral diencephalon in a pattern consistent with regulation of vessel sprouting at the midline. Several other known regulators of angiogenesis and axon growth are instead expressed in the ventral diencephalon, in particular *Slit1* and *Slit2*, which border the presumptive chiasm to contain RGC axons in a narrow corridor for commissure formation (Erskine et al., 2000; Plump et al., 2002). However, these molecules are unlikely candidates to prevent midline vessel ingression at the presumptive chiasm, because we observed that loss of Slit1 and Slit2 does not promote ectopic vessel sprouting in this area, even though it results in aberrant axon growth (Plump et al., 2002). Alternative factors with dual functions in blood vessel patterning and axon guidance are class 3 semaphorins, netrin 1 and SHH; however, none of these factors is expressed within the diencephalon midline (Deiner and Sretavan, 1999; Erskine et al., 2011; Torres et al., 1996). Beginning around E12.5, the vascular patterning cue *Efnb2* is expressed within the presumptive chiasmatic region to promote the ipsilateral projection of RGC axons (Williams et al., 2003). However, the midline region is avascular prior to this time, whereas surrounding diencephalic areas are well vascularised. Another signal for RGC axons that localises to the ventral diencephalic midline is SEMA6D (Kuwajima et al., 2012), but a role in modulating angiogenesis has not been reported previously. Further work will therefore be required to establish the importance of inhibitory signalling in regulating vessel sprouting into the ventral diencephalic midline.

### Conclusions

Through the analysis of tissue-specific knockouts, we have shown here that NRP1 has a dual role in patterning optic pathway development by acting directly in RGC neurons as an axon guidance receptor that promotes commissural axon sorting and indirectly by regulating neurovascular co-patterning through its role in endothelial cell signalling. Disrupting VEGF-A gradient formation also impacted indirectly on optic tract morphology through defective blood vessel patterning, highlighting the importance of normal vascular development for correct axon tract formation.

## MATERIALS AND METHODS

### Animals

Animal procedures were performed in accordance with institutional Animal Welfare and Ethical Review Bodies (AWERB) and UK Home Office guidelines. Mice were mated and the morning of vaginal plug formation counted as E0.5. Embryos were fixed overnight in 4% formaldehyde in PBS or used as donors for tissue culture experiments. We used the following mouse strains: wild-type C57BL/6J mice; mice carrying a *Nrp1* null allele on a mixed CD1:JF1 background that extends embryonic survival to E15.5 (Schwarz et al., 2004); *Nrp1^fl/fl^* mice carrying the *Rosa26^Yfp^* reporter crossed to mice carrying a heterozygous *Nrp1* null allele together with a *Tie2-Cre* (Fantin et al., 2013), *Six3-Cre* (Furuta et al., 2000) or *Nes-Cre* (Tronche et al., 1999) transgene or to mice carrying a *Brn3b^Cre^* knock-in allele (Simmons et al., 2016), all on a C57BL/6J background; mice carrying the *Vegfa^120^* or *Vegfa^188^* knock-in alleles (Ruhrberg et al., 2002), all on a C57BL/6J background; and *Slit1^−/−^* and *Slit1^−/−^;Slit^−/−^* compound mutants on a mixed C57BL/6J:129/Sv background (Plump et al., 2002). The *Brn3b^Cre^* allele was generated by homologous recombination in mouse embryonic stem cells using analogous strategies to the ones used previously to generate the *Brn3b^CKOAP^* alleles (Badea et al., 2009). For gene targeting, a vector was used in which a *PLAP-Neo* cassette in the first exon of *Brn3b* was replaced with a *Cre-Neo* cassette that contained a preceding IRES sequence.

### Anterograde and retrograde DiI labelling

Anterograde DiI labelling and calculation of the ipsilateral index was performed using fixed tissue as described previously (Erskine et al., 2011; Plump et al., 2002). Briefly, a crystal of DiI (Life Technologies, D282) was placed over the optic disc of one retina and the embryos were left at 37°C in PBS for 3-4 days. The brains were dissected and imaged, ventral side up or laterally, using a Nikon SMZ1500 microscope with a Nikon DXM1200 digital camera. The fluorescence intensity was determined in defined areas of the ipsilateral and contralateral optic tracts of non-saturated images using ImageJ (https://imagej.nih.gov/ij/). The ipsilateral index was calculated as the ratio of fluorescence intensity in the ipsilateral optic tract relative to the sum of the fluorescence intensity in the ipsilateral and contralateral optic tracts (Fig. S1A). Quantification of the number of optic pathway holes was performed using non-saturated images in a 300 µm-wide region at the midline of the ventral diencephalon and proximal 900 µm of the contralateral optic tract (Fig. S1B).

Retrograde labelling was performed as described previously (Erskine et al., 2011). Briefly, the cortex was removed on one side of formaldehyde-fixed brains and small crystals of DiI placed in a row over the dorsal thalamus. The fixed tissue was left at room temperature for 12-16 weeks before the ipsilateral and contralateral retina were flat-mounted and photographed as described above. The number of all labelled cells in the ipsilateral and contralateral retinas was determined by manual counting, and the percentage of labelled ipsilateral relative to the total number of labelled RGCs in both the ipsilateral and contralateral retina calculated. Analyses were performed blind to genotype.

### Immunofluorescence

Formaldehyde-fixed tissue was cryopreserved in 30% sucrose in PBS and embedded in OCT before snap freezing for cryosectioning at 15 µm. Alternatively, fixed tissue was embedded in 3% agarose and sectioned at 100 µm on a vibratome. Staining was performed as described (Thompson et al., 2009, 2006b). Primary antibodies used were: rabbit anti-neurofilament M (1:250; Millipore, AB1987), mouse anti-neuronal class III β-tubulin (TUJ1, also known as TUBB3; 1:1000; Cambridge Bioscience, MMS-435P) and goat anti-NRP1 (1:100; R&D Systems, AF566). Secondary antibodies were: Cy3-conjugated goat anti-rabbit IgG or anti-mouse IgG and Cy3-conjugated donkey anti-goat Fab fragment (Jackson ImmunoResearch). No staining was detected using the anti-NRP1 antibody in *Nrp1* null tissue (Fig. 2A,B; Fig. 3A,B; Figs S3 and S4). Blood vessels were detected using biotinylated IB4 (Sigma-Aldrich) followed by Alexa-Fluor633- or Alexa-Fluor488- conjugated streptavidin (Life Technologies). For combined DiI and IB4 labelling, detergents were omitted from all solutions. Images were captured using a Nikon SMZ1500 microscope, Zeiss Axiophot microscope or Zeiss LSM 510 or 710 confocal microscopes. Three-dimensional surface rendering of confocal *z*-stacks (optical slice depth, 1 µm; size of *z*-stacks 45-50 µm) was performed with Imaris (Bitplane). Quantification of vessel morphology was performed using ImageJ in confocal *z*-stacks. Analyses were performed blind to genotype.

### *In situ* hybridisation

*In situ* hybridisation was performed on 100 µm vibratome sections as described (Thompson et al., 2009, 2006a) using digoxigenin-labelled riboprobes for *Vegfa*, *Flt1*, *Slit1* or *Slit2* (Erskine et al., 2011, 2000). Images were captured using a Nikon SMZ1500 stereomicroscope with Nikon DS-Fi1c digital camera.

### Retina and aortic ring cultures

Peripheral regions of E14.5 wild-type mouse retinas were cultured in a 1:1 mixture of bovine dermal collagen and rat tail collagen as described (Erskine et al., 2011, 2000), except that we used MV2 media with endothelial cell growth supplement (Promocell) before (control) and after  (conditioned media) 72 h incubation with human microvascular endothelial cells (Promocell). After 48 h, the cultures were fixed and stained with antibodies for β-tubulin (Sigma-Aldrich, T8660; 1:500) followed by Cy3-conjugated goat anti-mouse IgG (1:2000). The area covered by RGC axons was quantified using ImageJ as described (Erskine et al., 2011, 2000). Analyses were performed blind to condition. Data are the mean (±s.e.m.) from three or more independent experiments.

Aortic rings were prepared as described (Therapontos et al., 2009) and cultured in 4-well plates at a distance of 100-300 µm from retinal explants in a mixture of DMEM (Life Technologies), rat tail collagen (3 mg/ml; BD Biosciences) and collagen type IV (30 µg/ml; BD Biosciences) in endothelial cell growth medium supplemented with the EGM-2 Bullet Kit (Lonza). Cultures were fixed after 48 or 96 h and stained with rabbit anti-neurofilament M and biotinylated IB4 followed by Cy3-conjugated goat anti-rabbit IgG and Alexa-Fluor488-conjugated streptavidin. Retinal axon outgrowth was quantified as described above. Confocal *z*-stacks were captured using a Zeiss LSM 710 confocal microscope and 3D reconstructions of the *z*-stacks generated using Imaris.

### Statistical tests

All data sets were analysed using the Shapiro–Wilk normality test. For normally distributed data, comparisons of two groups were made using Student's unpaired, two-tailed *t*-test, and comparisons of more than two groups by one-way ANOVA with Tukey post-hoc analysis. For data that did not appear normally distributed, comparisons were made using Kruskal–Wallis rank sum test with Tukey post-hoc analysis. The specific tests used are indicated in the figure legends. Because of background stain differences, direct statistical comparisons were not made between mutations on different genetic backgrounds.
